# Pulmonary tuberculosis screening in anti-retroviral treated adults living with HIV in Kenya

**DOI:** 10.1186/s12879-021-05916-z

**Published:** 2021-02-25

**Authors:** Jill K. Gersh, Ruanne V. Barnabas, Daniel Matemo, John Kinuthia, Zachary Feldman, Sylvia M. Lacourse, Jerphason Mecha, Alex J. Warr, Maureen Kamene, David J. Horne

**Affiliations:** 1 Department of Medicine, Division of Allergy and Infectious Diseases, University of Washington, Seattle, WA USA; 2 Department of Global Health, University of Washington, 325 9th Ave, Box 359762, Seattle, WA 98102 USA; 3 Department of Epidemiology, University of Washington, Seattle, WA USA; 4 Department of Obstetrics and Gynaecology, Kenyatta National Hospital, Nairobi, Kenya; 5 School of Public Health and Community Development Maseno University, Kisumu, Kenya; 6 Albers School of Business and Economics, Seattle University, Seattle, WA USA; 7 Department of Pediatrics, Department of Medicine, Baylor College of Medicine, Houston, TX USA; 8 National Tuberculosis, Leprosy, and Lung Disease Program, Nairobi, Kenya; 9 Department of Medicine, Division of Pulmonary, Critical Care, and Sleep Medicine, University of Washington, Harborview Medical Center, Seattle, WA USA

**Keywords:** Tuberculosis, Latent Tuberculosis, AIDS-related opportunistic infections epidemiology, Diagnostic tests, Prevalence

## Abstract

**Background:**

People living with HIV (PLHIV) who reside in high tuberculosis burden settings remain at risk for tuberculosis disease despite treatment with anti-retroviral therapy and isoniazid preventive therapy (IPT). The performance of the World Health Organization (WHO) symptom screen for tuberculosis in PLHIV receiving anti-retroviral therapy is sub-optimal and alternative screening strategies are needed.

**Methods:**

We enrolled HIV-positive adults into a prospective study in western Kenya. Individuals who were IPT-naïve or had completed IPT > 6 months prior to enrollment were eligible. We evaluated tuberculosis prevalence overall and by IPT status. We assessed the accuracy of the WHO symptom screen, GeneXpert MTB/RIF (Xpert), and candidate biomarkers including C-reactive protein (CRP), hemoglobin, erythrocyte sedimentation rate (ESR), and monocyte-to-lymphocyte ratio for identifying pulmonary tuberculosis. Some participants were evaluated at 6 months post-enrollment for tuberculosis.

**Results:**

The study included 383 PLHIV, of whom > 99% were on antiretrovirals and 88% had received IPT, completed a median of 1.1 years (IQR 0.8–1.55) prior to enrollment. The prevalence of pulmonary tuberculosis at enrollment was 1.3% (*n* = 5, 95% CI 0.4–3.0%): 4.3% (0.5–14.5%) among IPT-naïve and 0.9% (0.2–2.6%) among IPT-treated participants. The sensitivity of the WHO symptom screen was 0% (0–52%) and specificity 87% (83–90%). Xpert and candidate biomarkers had poor to moderate sensitivity; the most accurate biomarker was CRP ≥ 3.3 mg/L (sensitivity 80% (28–100) and specificity 72% (67–77)). Six months after enrollment, the incidence rate of pulmonary tuberculosis following IPT completion was 0.84 per 100 person-years (95% CI, 0.31–2.23).

**Conclusions:**

In Kenyan PLHIV treated with IPT, tuberculosis prevalence was low at a median of 1.4 years after IPT completion. WHO symptoms screening, Xpert, and candidate biomarkers were insensitive for identifying pulmonary tuberculosis in antiretroviral-treated PLHIV.

## Introduction

Tuberculosis (TB) remains the leading cause of death in people living with HIV (PLHIV). TB preventive therapy is a critical intervention with mortality benefits that are independent of antiretroviral therapy (ART) [1, 2]. Isoniazid preventive therapy (IPT) administered for 6 months is a World Health Organization (WHO) recommended regimen that remains the most widely used TB preventive therapy [3]. In moderate and high TB burden areas, re-infection with *M. tuberculosis* due to frequent re-exposure may limit the durability of TB preventive therapy’s protective benefit. This was observed in the initial trials of IPT in PLHIV [4–6], in which TB rates significantly increased approximately 6 months after completing treatment. Recent trials suggest that IPT’s protective effects may be more durable when administered to antiretroviral-treated PLHIV [1, 7]. Little is known about the effectiveness of IPT in high TB burden settings outside of research trials, where pre-IPT screening for TB may include sputum evaluations and/or chest radiographs [5–8], and adherence may be optimized.

WHO recommends a symptom-based intensified case finding (ICF) screen to determine PLHIV who are eligible for immediate TB preventive treatment and as a general triage test at clinic visits to determine who needs further evaluation for TB disease [9]. Notably, this ICF screen was developed and validated in PLHIV who were new to HIV care and therefore ART-naive [10]. Subsequent studies have found that the WHO ICF screen has poorer sensitivity in identifying TB in ART-treated individuals [11]. As this screen is being used to evaluate for TB in ART-treated individuals (regardless of IPT status), it is important to understand its performance and limitations under programmatic conditions. Furthermore, there is an urgent need for novel strategies to risk stratify individuals for TB evaluations. A WHO consensus-gathering meeting identified a rapid, non-sputum-based diagnostic test that accurately diagnoses pulmonary TB as the most urgently needed TB test, and established a target product profile of sensitivity greater than 90% and specificity greater than 70% [12].

In 2017, 67 countries reported providing TB preventive treatment to nearly 1 million PLHIV [13]. However, provision of TB preventive treatment remains poor, with estimated coverage of only 36% of eligible PLHIV [13]. Kenya is one of four HIV high-burden countries in Africa [14], and one of the twenty highest TB burden nations with an estimated overall TB incidence of 348/100,000 person-years in 2016 [15]. In 2015, Kenya Ministry of Health initiated provision of IPT (6-month course) to PLHIV and provided IPT to almost 400,000 PLHIV in 2016, among the highest in the world [16]. We developed the current pilot study to investigate knowledge gaps around TB prevention in PLHIV including the prevalence of TB in PLHIV who received IPT under programmatic conditions in the setting of widespread ART availability and the performance of established and novel TB screening tools. We evaluated the accuracy of the WHO ICF screen for TB and investigated the performance of candidate biomarkers as TB triage tests in PLHIV who were overwhelmingly receiving ART.

## Methods

### Study setting

Between March 2017 and September 2018, we performed a prospective study among PLHIV at two HIV-care clinics in western Kenya.

### Participants

PLHIV ages 18 to 70 years were eligible for study enrollment if they were either IPT-naïve or had completed IPT at least 6 months prior to enrollment. Individuals who were unable to provide consent in a study language (English or Dholuo), pregnant, incarcerated, or were unwilling to provide a home location were ineligible for participation.

### Procedures

#### Enrollment

We recruited PLHIV following an outpatient HIV clinic visit in western Kenya. Due to enrollment constraints, one participant per day (usually the first eligible patient seen that day) was enrolled at each site. After providing informed consent (written or, if illiterate, the consent was read and understanding confirmed with a thumb print), participants were interviewed by study staff using a structured interview tool that included questions on sociodemographic information, HIV history, and TB and IPT histories. Data extracted from clinic charts included results of TB screening on the day of enrollment, medication history, and laboratory values including CD4 cell count and HIV viral load. Phlebotomy was performed for complete blood count (CBC) with differential, hemoglobin, C-reactive protein (CRP), erythrocyte sedimentation rate (ESR), and HIV viral load (if not available from the clinic chart within the prior 6 months). Study data were collected and managed using REDCap electronic data capture tools [17].

#### Tuberculin skin test (TSTs)

TSTs were performed by study personnel using 5 tuberculin units (0.1 ml) of purified protein derivative (RT23 solution, Sanofi Pasteur) and read using the ball-point technique and a ruler within 48–96 h [18, 19]. A positive TST was defined as > 5 mm of induration [20].

#### Sputum collection and TB laboratory testing

Participants were instructed on sputum collection and a “spot” sample was collected at the time of enrollment. If this was unsuccessful, then the participant was provided with a collection container and instructed to collect an early morning specimen upon awakening on the day of TST read. At the enrollment sites, sputum samples were refrigerated and transported same day on ice at 4–8 °C to an ISO 15189-accredited laboratory. Specimens were decontaminated using *N*-acetyl-L-cysteine and sodium hydroxide and examined using fluorescence microscopy. If one or more acid-fast bacilli (AFB) per equivalent of 100 immersion fields was observed, the slide was considered positive and graded. After re-suspension with phosphate buffer, equal sample volumes were used to perform mycobacterial culture and GeneXpert MTB/RIF (Xpert, Cepheid, Sunnyvale, CA). Mycobacterial culture was performed using a commercial broth method, MGIT Manual Mycobacterial Growth System (Becton-Dickinson, Franklin Lakes, NJ). The Xpert assay assigns a semiquantitative category to positive tests for *M. tuberculosis* based on cycle threshold (Ct) values: “high,” Ct ≤ 16; “medium,” 16 < Ct ≤ 22; “low,” 22 < Ct ≤ 28; and “very low,” 28 < Ct ≤ 38 [21]. Isolates were identified as *M. tuberculosis* using the Capilia TB Test Kit (TAUNS, Numazu, Japan). All smear, culture and initial Xpert tests were performed on fresh samples. In the event of a positive Xpert with negative culture, Xpert was re-performed on the frozen sample.

#### Candidate biomarkers

Candidate biomarkers included CRP, hemoglobin, ESR, monocyte-to-lymphocyte ratio (MLR), and the neutrophil-to-lymphocyte ratio (NLR). Investigators and clinicians were unaware of laboratory test results when assessing participants for TB disease. CRP levels were measured using a high sensitivity assay (Cobas Integra 400 Plus (Roche Diagnostics, Rotkreuz, Switzerland). HIV viral load testing was performed using the COBAS AmpliPrep/COBAS TaqMan HIV-1 Qual test (Roche Diagnostics). CBC with differential was performed using a Coulter ACT 5Diff CP analyzer (Beckman Coulter, France). The MLR and NLR were calculated as the absolute monocyte count or absolute neutrophil count divided by the absolute lymphocyte, respectively.

### Six-month follow-up

Study participants were invited back 6 months after enrollment for additional data collection and repeat evaluations for TB. Due to study closure in September 2018, only participants who were enrolled prior to March 2018 were eligible for 6-month follow-up evaluations. Evaluations included a questionnaire, review of medical records, sputum collection for AFB-smear and -culture, and phlebotomy.

### Study endpoints and statistical analysis

At enrollment, pulmonary TB was defined as at least one sputum culture positive for *M. tuberculosis*. At least one valid negative AFB-culture result was required to define participants as not having pulmonary TB (e.g. participants with missing data or only one culture result that was contaminated were excluded from analyses). Analyses were performed using Stata 14 (StataCorp, College Station, TX). TB incidence rates were calculated for participants with a history of IPT using the Stata command “stptime” with failure defined as TB diagnosis (culture-based or clinical diagnosis). Observation time was defined as time from IPT completion until the 6-month follow-up visit. If participants were not seen at 6-month follow-up, observation time was the time from completing IPT to study enrollment. Bivariate logistic regression and Fisher’s exact test were used to assess the association between potential correlates and the outcome of pulmonary TB. 95% confidence intervals (CI) were derived using logistic regression. Distributions of candidate biomarkers were compared by TB status using the Kruskal-Wallis test. The performance of the WHO ICF algorithm, AFB-smear, Xpert, and candidate biomarkers were compared to AFB-culture using sensitivity, specificity, and positive and negative predictive values. Receiver operating characteristic (ROC) curves of candidate biomarkers were determined using the “roctab” command in Stata and asymptotic normal CIs calculated using a published algorithm [22]. Optimal diagnostic cutoffs were determined based on the maximum value of Youden’s index (i.e. sensitivity + specificity – 1) [23]. All statistical tests were two-sided with α = 0.05.

We set a target enrollment of 400 participants based on an estimate that one-half of participants would be ART-naïve with a TB prevalence of 11–12% [24], and that TB prevalence among ART-treated participants would be ~ 3% [25]. An enrollment goal of 400 participants would support the evaluations of screening tests that met the WHO target product profile (90% sensitivity, 70% specificity) at α = 0.05 and establish TB prevalence estimates with error margins of + 2.5% [26].

### Ethics approval

This study was approved by the Kenyatta National Hospital-University of Nairobi Ethics and Research Committee and the University of Washington Institutional Review Board.

## Results

Between March 2017 and June 2018, 390 PLHIV were screened for study eligibility, among whom two declined study entry, two did not have sputum collected, and three had contaminated sputum cultures. Among 383 participants included in the analysis, the median age was 37 years (interquartile range (IQR) 31–45) (Table 1). Among participants with known ART status (*n* = 380), all but one participant (99.7%) were taking ART at the time of study enrollment, with a median time on ART of 5.8 years (IQR 2.4–9.2). HIV viral load within the 6 months prior to study enrollment was available for 372 participants with a median value of 20 copies/mL (IQR 0–41) and 93.0% were viral load suppressed by Kenyan Ministry of Health guidelines (< 1000 copies/mL) [27]. A history of TB was present in 15.3% of participants. TST results were available on 330 participants, 21.5% of whom had induration ≥ 5 mm.

**Table 1:** Participant characteristics overall and by TB status. N (%) unless otherwise indicated

Characteristics	All (***n*** = 383)	TB (***n*** = 5)	No TB (***n*** = 378)	OR	95% CI‖	***p***-value
**Sociodemographic**						
Age, years (median (IQR))	37 (31–45)	32 (30–32)	37 (31–45)	0.96	(0.87–1.06)	0.37
Men	159 (41.5)	1 (20.0)	158 (41.8)	0.35	(0.04–3.14)	0.35
BMI (median (IQR))	22.1 (19.7–24.8)	23.4 (21–26)	22.1 (19.7–24.8)	1.00	(0.98–1.03)	0.79
Education, years (median (IQR))	8 (7–12)	8 (7–10)	8 (7–12)	1.05	(0.79–1.40)	0.74
Unemployed	60 (15.7)	0	60 (15.9)			0.33
Currently married	261 (68.2)	5 (100)	256 (67.7)			0.12
Alcohol use, current (*n* = 377)	49 (12.7)	0 (0)	48 (12.7) (*n* = 377)			0.39
**IPT**						
IPT history (*n* = 381)	330 (86.6)	3 (60.0)	327 (87.0)^a^	0.22	(0.04–1.38)	0.11
Completed 6 months of IPT (*n* = 328)	317 (96.7)	3 (100) *n* = 3	314 (96.6)^b^			0.75
**HIV**						
ART, current (*n* = 380)	379 (99.7)	5 (100)	374 (99.7)^c^ (*n* = 375)			0.91
TDF/3TC/EFV (*n* = 377)	182 (48.3)	3 (60)	179 (48.1) (*n* = 372)			
TDF/3TC/NVP	124 (32.9)	1 (20)	123 (33.1)			
AZT/3TC/NVP	34 (9.0)	1 (20)	33 (8.9)			
TDF/3TC/LPVr	7 (1.9)	0	7 (1.9)			
AZT/3TC/LPVr	7 (1.9)	0	7 (1.9)			
Other/unknown	23 (6.1)	0	23 (6.2)			
Time on ART, years (median (IQR)) (*n* = 378)	5.76 (2.39–9.19)	3.76 (1.31–3.85)	5.82 (2.39–9.20)^d^	0.98	(0.93–1.04)	0.53
CD4 (*n* = 314) (median (IQR))	405 (276–558)	370 (195–398)	408 (277–558) (*n* = 309)	1.00	(0.99–1.00)	0.35
CD4 < 200 cells/mm^3^	43 (13.7)	2 (40)	41 (13.3)	4.36	(0.71–26.9)	0.11
CD4 < 500 cells/mm^3^	209 (66.6)	4 (80)	205 (66.3)	2.03	(0.23–18.4)	0.53
VL, IU/mL (*n* = 372)	20 (0–41)	9 (0–20)	20 (0–50) (*n* = 367)	1.00	(0.99–1.0)	0.72
Suppressed VL, < 1000 IU/mL	346 (93.0)	5 (100)	341 (92.9)			0.54
Co-trimoxazole	362 (94.5)	5 (100)	357 (94.4)			0.59
**TB symptoms**						
Any (*n* = 382)	48 (12.6)	0 (0)	48 (12.7) (*n* = 377)			0.39
Current cough	31 (8.0)	0	31 (8.1)			
Cough > 2 weeks	8 (2.1)	0	8 (2.1)			
Weight loss	9 (2.3)	0	9 (2.4)			
Fever	5 (1.3)	0	5 (1.3)			
Night sweats	18 (4.7)	0	18 (4.8)			
TB symptoms present in clinic chart (*n* = 267)	5 (1.9)	0	5 (1.9) (*n* = 261)			0.76
History of TB (*n* = 380)	58 (15.3)	2 (50) (*n* = 4)	56 (14.9)^a^	5.71	(0.79–41.4)	0.09
TST, mm induration (*n* = 330)	0 (0–4)	6 (0–17)	0 (0–4)	1.18	(1.01–1.38)	0.03
TST ≥ 5 mm (*n* = 330)	72 (21.5)	2 (50) (*n* = 4)	70 (21.5) (*n* = 326)	3.66	(0.5–26.4)	0.20
TST ≥ 10 mm (*n* = 330)	38 (11.5)	2 (50) (*n* = 4)	36 (11.0) (*n* = 326)	8.06	(1.10–58.95)	0.04
History of TB in household members (*n* = 381)	42 (10.9)	1 (20)	40 (10.6) (*n* = 376)	2.10	(0.23–19.25)	0.51

*OR* Odds ratio, *CI* Confidence interval, *IQR* Inter-quartile range, *BMI* Body mass index, *IPT* Isoniazid preventive therapy, *TDF* Tenofovir disoproxil fumarate, *3TC* Lamivudine, *EFV* Efavirenz, *NVP* Nevirapine, *AZT* Zidovudine, *LPVr* Lopinavir/ritonavir, *CD4* CD4 cell count, *VL* Viral load, *TST* Tuberculin skin test; ‖95% CIs based on logistic regression estimates; ^a^*n* = 376; ^b^*n* = 325; ^c^ *n* = 375; ^d^*n* = 373;

### Sputum results

Sputum culture results were available for 383 participants, 376 collected as spot samples and seven collected by participants on awakening at home. All sputum AFB-smears were negative. Sputum samples from 5 participants, all collected as spot specimens, were culture-positive for *M. tuberculosis*. The prevalence of pulmonary TB was 1.3% (95% CI 0.4–3.0%). Xpert was initially reported as positive in 4 samples, with semiquantitative grading of low in one sample and very low in three samples. Only the sample graded as low was culture-positive for *M. tuberculosis*. Xpert testing was repeated in the 3 culture-negative (very low) samples and all were Xpert negative on repeat testing of the frozen sample. There were no indeterminate results or rifampin resistance detected by Xpert.

### IPT and TB

IPT history was available for 381 participants, of whom 339 (87.7%) had received IPT. There were significant differences in baseline characteristic (Table 1) between participants by IPT history for current use of ART (2% ART-naïve among IPT-naïve participants vs. 0% in IPT-treated, *p*-value = 0.01), median time on ART (0.13 years vs. 6.7 years, *p*-value < 0.001), median CD4 cell count (342 cells/mm^3^ vs. 413 cells/mm^3^, *p*-value = 0.01), and viral suppression (78.3% vs. 95.1%, *p*-value < 0.001). No participants had been diagnosed with TB since completing IPT. In 328 participants for whom the date of IPT completion was available, the median time between IPT completion and study enrollment was 1.1 years (IQR 0.8–1.5) and 96.7% reported completing a full 6-month course. Pulmonary TB was diagnosed in two of 47 IPT-naïve participants (4.3, 95% CI 0.5–14.5%) and three of 334 IPT-treated participants (0.9, 95% CI 0.2–2.6%). Two of the five participants with TB disease had a prior history of treated TB, both of whom had received IPT. Isoniazid-monoresistant TB was diagnosed in one participant, who had previously completed treatment with IPT 19 months prior to study enrollment and had a history of TB disease 12 years prior to enrollment. Prior IPT history was not significantly associated with TB disease (OR 0.22, 95% CI 0.04–1.38, *p*-value 0.11).

### Association of TB with predictors

We assessed associations between candidate predictors and TB disease (Table 1). TST ≥ 10 mm was the only predictor that was significantly associated with TB disease (OR 8.1, 95% CI 1.1–59.0, p-value 0.04).

### Accuracy of WHO ICF algorithm and candidate biomarkers for diagnosis of TB

The results of a symptom screen performed during a programmatic clinic visit on the same day as study enrollment were available for 267 participants. Clinic-based screening identified 5 participants (1.9%) with a positive WHO ICF screen, 3 of whom were also found to have symptoms during the study enrollment process (kappa = 0.13). When administered by study personnel, the WHO ICF algorithm identified 48 (12.6%) participants with TB symptoms, none of whom had a positive culture for *M. tuberculosis*. Overall, the WHO ICF algorithm performed by study staff had sensitivity of 0% (95% CI 0–52%), specificity 87% (95% CI 83–90%), positive predictive value 0% (95% CI 0–7%), and negative predictive value 99% (95% CI 97–100%) for identifying pulmonary TB. The initial Xpert result was 20% sensitive (95% CI 1–72%), 99% specific (95% CI 98–100%), positive predictive value of 25% (95% CI 1–81%), and negative predictive value of 99% (95% CI 97–100%) for identifying pulmonary TB.

We evaluated additional candidate biomarkers for TB disease. Various cut-points for the biomarkers were obtained from published studies, including CRP [28–30], hemoglobin [31, 32], and monocyte-to-lymphocyte ratio [33], and/or derived from the current study based on Youden’s index. (Table 2) ESR and CRP had the largest areas under the ROC curve. In our study, the use of published cutoffs for CRP at 5 mg/L [30], 8 mg/L [29], and 10 mg/L [28]; hemoglobin at 8 g/dL [31]; and MLR at 0.285 [33]; were associated with poor sensitivity and PPV. In terms of optimizing sensitivity and specificity, the biomarker and cut-point that was closest to the WHO target product profile [12] was CRP > 3.3 mg/L: sensitivity 80% (95% CI, 28–100), specificity 72% (95% CI, 67–77).

**Table 2:** Test and biomarker performance in identifying tuberculosis. % (95% CI)

Test (***n*** = 382)	Sensitivity	Specificity	PPV	NPV	AUROC (95% CI)	Youden’s index
**Any TB symptom**	0 (0–52)	87 (84–91)	0 (0–7)	99 (97–100)	0.436 (0.419–0.453)	
**GeneXpert**	20 (1–72)	99 (98–100)	25 (1–81)	99 (97–100)	0.596 (0.400–0.792)	
**TST, induration**					0.629 (0.244–1.00)	
≥ 5 mm	50 (7–93)	79 (74–83)	3 (0–10)	99 (97–100)		
≥ 10 mm	50 (7–93)	89 (85–92)	5 (1–18)	99 (98–100)		
**CRP**					0.683 (0.355–1.00)	
> 3.3 mg/L	80 (28–100)	72 (67–76)	4 (1–9)	100 (98–100)		0.518
> 5 mg/L	40 (5–85)	79 (75–83)	3 (0.3–9)	99 (97–100)		0.193
> 8 mg/L	40 (5–85)	89 (85–92)	4 (1–15)	99 (97–100)		0.286
> 10 mg/L	20 (1–72)	90 (86–93)	3 (0–14)	99 (97–100)		0.099
**Hemoglobin**					0.352 (0.027–0.676)	
< 8 g/dL	0 (0–52)	94 (91–96)	0 (0–15)	99 (97–100)		−0.064
< 10 g/dL	60 (15–95)	86 (82–89)	6 (1–16)	99 (98–100)		0.458
< 15.3 g/dL	80 (28–100)	12 (9–16)	1 (0–3)	98 (88–100)		0.082
**MLR (*n*** **= 362)**					0.449 (0.141–0.757)	
> 0.208	40 (5–85)	81 (76–85)	3 (0–10)	99 (97–100)		0.207
≥ 0.285	0 (0–52)	94 (91–96)	0 (0–16)	99 (97–100)		−0.059
**NLR (*n*** **= 360)**					0.540 (0.200–0.879)	
≥ 1.5	40 (5–85)	80 (76–84)	3 (0–10)	99 (97–100)		0.200
**ESR (*n*** **= 380)**					0.682 (0.436–0.927)	
≥ 35.5 mm	60 (15–95)	78 (74–82)	4 (1–10)	99 (99–100)		0.381

*TB* Tuberculosis, *TST* Tuberculin skin test, *CRP* C-reactive protein, *MLR* Monocyte to lymphocyte ratio, *NLR* Neutrophil to lymphocyte ratio, *ESR* Erythrocyte sedimentation rate, *PPV* Positive predictive value, *NPV* Negative predictive value, *AUROC* Area under the receiver operating characteristic curve - ROC and 95% CIs estimates derived using “roctab” command in Stata [22]

### Follow-up

205 participants were seen at six-month follow-up, four of whom had contaminated sputum cultures. There were significant differences in baseline characteristic (Table 1) between participants seen at six-month follow-up compared to those not seen in follow-up for any symptoms (present in 7.1% with follow-up and 19.2% without follow-up, *p*-value = 0.001), current cough (2.8% vs. 14.5%, *p*-value < 0.001), and employment status (9.0% vs 23.8%, *p*-value < 0.001). The median follow-up time among the included 201 participants was 195 days (IQR 175–230). 11.6% of participants at follow-up reported at least one symptom consistent with TB; only 2.5% of participants seen at 6 months reported at least one symptom consistent with TB at both enrollment and follow-up. One participant was diagnosed with TB 3 days prior to the follow-up visit. This was a clinical diagnosis after the participant presented with symptoms of cough, fever, night sweats and 3 kg weight loss. Among the 201 participants, no sputum samples were positive for *M. tuberculosis*. Among all study participants who had received IPT (*n* = 339), the median follow-up time was 1.4 years (IQR 1.22–1.6). Over 478 person-years of observation, the incidence rate of TB following IPT completion (*n* = 4) was estimated as 0.84 per 100 person-years (95% CI, 0.31–2.23).

## Discussion

We investigated the prevalence of pulmonary TB in PLHIV receiving antiretroviral therapy, the majority of whom (88%) had completed IPT at least 6 months prior to study enrollment. We found that IPT-treated participants had high self-reported levels of completion and low annual risk of TB disease. The efficacy of IPT in preventing TB has been well-demonstrated [1, 2, 5–7, 34]. In our study, the prevalence of pulmonary TB at enrollment in IPT-naïve participants was 4.3% (95% CI 0.5–14.5%) and in IPT-treated participants was 0.9% (95% CI 0.2–2.6%). Our study did not identify an association between receipt of IPT and decreased frequency of TB (OR 0.22, 95% CI 0.04–1.38), although this was likely due to lack of statistical power. TB prevention trials that were performed in PLHIV not receiving antiretroviral therapy, were notable for the limited duration of protection against TB disease afforded by 6 months of isoniazid, with mixed results on the efficacy of long-term (i.e., 36 months) isoniazid treatment [4–6, 34]. A long-term follow-up of the TEMPRANO study of IPT and early ART, demonstrated that the benefits of 6 months of isoniazid, including mortality benefits, were sustained for 6 years in the context of concurrent antiretroviral therapy treatment [1]. The median time since IPT completion in our study was 1.4 years, precluding an evaluation of long-term IPT durability.

One cited reason for low global uptake of IPT has been concern over the potential for selection for drug resistance when isoniazid is administered to patients with unrecognized TB disease [35]. One of the five participants diagnosed with culture-positive TB in our study was found to have isoniazid-mono-resistant disease. This participant had a history of both TB disease and IPT-treatment. Our single participant with isoniazid-resistant TB may reflect the background level of isoniazid monoresistance in western Kenya, approximately 5% [36, 37], although we cannot rule-out that this resistance may have been related to IPT. Although no published trials have demonstrated increased isoniazid resistance due to IPT [5, 38–40], these trials stringently evaluated for TB disease prior to IPT and it is not known whether IPT, when used under programmatic conditions, will increase the risk for isoniazid-resistant TB due to inadvertent treatment of individuals with subclinical TB disease.

We found that in participants diagnosed with culture-confirmed TB disease, various TB screens, including WHO ICF screen, Xpert, CRP, hemoglobin, and monocyte-to-lymphocyte ratio, performed poorly. WHO recommends a symptom-based ICF screen to stratify patients to TB preventive therapy or further evaluation for TB disease [9]. In our study, there were no symptomatic participants diagnosed with pulmonary TB. Prior studies have described the poor performance of symptom screen in antiretroviral treated individuals [25, 41, 42], and a recent meta-analysis estimated the pooled sensitivity and specificity of the symptom screen in ART-treated individuals as 51% (95% CI 28–73) and 71% (95% CI 48–86), respectively [11]. The poorer sensitivity of this screen in ART-treated individuals may due to greater immunocompetency predisposing to asymptomatic TB, or frequent TB screening reducing the frequency of individuals with advanced and symptomatic TB [11]. We believe that our study participants were subject to frequent screening for TB as this is recommended in the Kenyan national guidelines [43], and 70% of study participants were screened for TB during a clinic visit on the same day that they were enrolled in the study. However, differences in screening results between the clinic visit (2% with a positive symptom screen) and study screening (13% positive screen) highlight an additional limitation of the ICF screen, its high inter-rater variability.

Although four participants were initially found to be Xpert positive, we believe that three of these results were false positives based on AFB-culture negativity, very low semiquantitative grading, and negative repeat testing on the frozen sample [21]. Quantitative readouts have been previously used to guide investigation of potentially false-positive results. It is unlikely that use of frozen samples for re-testing would have led to a decrease in Xpert sensitivity [44, 45].

WHO issued guidance on target product profiles for community-based triage or referral tests to identify individuals suspected of having TB, suggested a sensitivity greater than 90% and specificity greater than 70% [12]. In our study, neither Xpert nor candidate biomarkers, achieved the product profiles suggested by WHO. A recent systematic review of Xpert accuracy when applied to TB suspects found pooled sensitivities of 67% (95% CI 62–72%) and 81% (95% CI 75–86%) for smear-negative disease and TB in PLHIV, respectively [46]. The poor sensitivity of Xpert was likely due, in part, to paucibacillary disease. CRP has performed well in a number of studies [28], including a study of PLHIV initiating antiretroviral therapy in Uganda that identified sensitivity of 90% and specificity 70% for CRP > 8 mg/L. [29] We did not replicate these findings in our study which applied these tests irrespective of signs or symptoms in antiretroviral-treated PLHIV subject to frequent TB symptoms screens.

Among study participants, 12.2% had not received IPT. Self-reported IPT 6-month completion (97%) and adherence (98%) were high, comparable to recent trial results [1]. As shorter course regimens for TB prevention in PLHIV are introduced [47, 48], it is anticipated that rates of adherence and completion will be high. The TB prevention cascade [49], modeled on the HIV care cascade, is a framework to understand where patient losses are occurring along the continuum of TB prevention. Addressing losses in the TB prevention care continuum may be most cost-effective if targeted at decreasing the number of PLHIV who do not start TB prevention regimens.

Our study has several limitations. We diagnosed few participants with TB disease which limited our ability to perform multivariable analyses due to a limited number of events (TB) per variable [50]. Although we performed sample size calculations prior to study initiation, we enrolled fewer ART-naïve participants than anticipated and TB prevalence was lower in ART-treated participants than estimated. This precluded the evaluation of screening algorithms that used a combination of tests. We collected one sputum sample from each participant, potentially leading to underestimation of the true TB burden in our study population. There were baseline differences between participants who had received IPT compared to those who had not for ART status, CD4 cell count and viral suppression, all of which could increase the risk for TB among IPT-naïve individuals. We did not prospectively assess IPT adherence and remote recall may be subject to bias [51]. We were unable to determine the durability of IPT’s protective effect against TB beyond approximately one-year post-IPT. There were also baseline differences between participants seen at six-month follow-up compared to those not seen at 6 months in the presence of any TB symptom and current cough, both of which were more frequent in participants not seen in follow-up. These differences could potentially have led to a bias of underdiagnosis of TB at the follow-up visit.

In conclusion, in Kenyan PLHIV we found that IPT-uptake was high and TB prevalence was low at a median of 1.4 years after IPT-completion. We also determined that symptom-based screening, Xpert, and candidate biomarkers were insensitive for identifying ART-treated PLHIV who should undergo evaluations for TB disease. Our study results suggest several future directions for research. Although IPT-uptake and reported adherence was high, understanding patients’ reasons for not initiating TB preventive therapy and interventions to increase acceptance and completion of preventive regimens will be important to improve outcomes across the TB prevention continuum. Importantly for PLHIV in high burden settings, the TB prevention cascade includes TB screening following preventive therapy, and potentially repeat administration of TB preventive therapy, as individuals are at risk for re-infection with *M. tuberculosis* and progression to TB disease [52]. Prospective studies of TB preventive therapy in PLHIV will l need to enroll large numbers of participants given the effectiveness of IPT in antiretroviral-treated individuals. Finally, triage tests for TB risk stratification will need to be carefully studied and validated by ART status.

## Data Availability

The datasets analyzed during the current study are available in the figshare repository (10.6084/m9.figshare.13514728).

## Abbreviations

AFB: Acid-fast bacilli
ART: Antiretroviral therapy
CBC: Complete blood count
CI: Confidence interval
CRP: C-reactive protein
Ct: Cycle threshold
ESR: Erythrocyte sedimentation rate
HIV: Human immunodeficiency virus
ICF: Intensified case finding
IPT: Isoniazid preventive therapy
IQR: Interquartile range
MLR: Monocyte-to-lymphocyte ratio
NLR: Neutrophil-to-lymphocyte ratio
PLHIV: People living with HIV
TB: Tuberculosis
TST: Tuberculin skin test
WHO: World Health Organization
